# District-level approach for tailoring and targeting interventions: a new path for malaria control and elimination

**DOI:** 10.1186/s12936-020-03185-w

**Published:** 2020-03-30

**Authors:** Roly Gosling, John Chimumbwa, Petrina Uusiku, Sara Rossi, Henry Ntuku, Kelly Harvard, Chris White, Allison Tatarsky, Daniel Chandramohan, Ingrid Chen

**Affiliations:** 1 Malaria Elimination Initiative, Global Health Group, University of California, San Francisco, CA 94158 USA; 2 Department of Epidemiology and Biostatistics, University of California, San Francisco, CA 94158 USA; 3 Multidisciplinary Research Centre, University of Namibia, Windhoek, Namibia; 4 Elimination 8, Channel Life Towers 1st Floor 39 Post Street Mall, Windhoek, Namibia; 5 National Vectorborne Disease Control Programme, Ministry of Health and Social Services, Private Bag 13198, Windhoek, Namibia; 6 London School of Hygiene and Tropical Medicine, Keppel Street, London, WC1E 7HT UK

**Keywords:** Malaria, Control, Elimination, Policy, Community engagement, Surveillance, District health management team, High risk populations, Residual transmission

## Abstract

Despite huge investments and implementation of effective interventions for malaria, progress has stalled, with transmission being increasingly localized among difficult-to-reach populations and outdoor-biting vectors. Targeting difficult pockets of transmission will require the development of tailored and targeted approaches suited to local context, drawing from insights close to the frontlines. Districts are best placed to develop tailored, locally appropriate approaches. We propose a reorganization of how malaria services are delivered. Firstly, enabling district health officers to serve as conduits between technical experts in national malaria control programmes and local community leaders with knowledge specific to local, at-risk populations; secondly, empowering district health teams to make malaria control decisions. This is a radical shift that requires the national programme to cede some control. Shifting towards a district or provincial level approach will necessitate deliberate planning, and repeated, careful assessment, starting with piloting and learning through experience. Donors will need to alter current practice, allowing for flexible funding to be controlled at sub-national levels, and to mix finances between case management, vector control and surveillance, monitoring and evaluation. System-wide changes proposed are challenging but may be necessary to overcome stalled progress in malaria control and elimination and introduce targeted interventions tailored to the needs of diverse malaria affected populations.

## Background

**Fig. 1:**
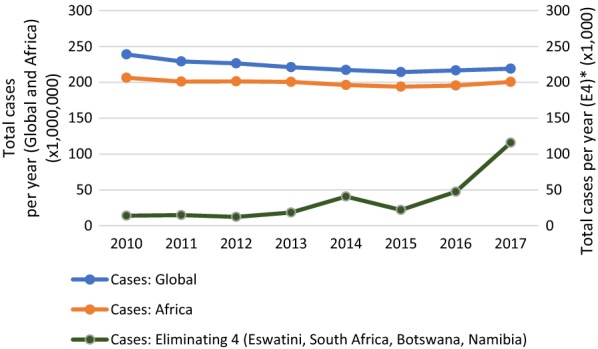
Reported malaria cases globally, in Africa, and in the four eliminating countries in southern Africa from 2010–2017. Reported malaria cases globally and in Africa, and in the 4 eliminating countries (Botswana, Eswatini, Namibia, South Africa– E4) of the Elimination 8 countries in southern Africa

Since 2000, substantial growth in international and domestic funding has facilitated a surge of global progress in the fight against malaria [1, 2]. National malaria control programmes (NMCPs) have scaled up a number of highly effective standard interventions, including malaria rapid diagnostic tests (RDTs), artemisinin-based combination therapy (ACT), long-lasting insecticide-treated bed nets (LLINs), and indoor residual spraying (IRS). This has led to significant reductions in malaria cases and deaths, until 2015 when progress began plateauing globally and even regressing in some countries, including several in southern Africa (Fig. 1).

Why is progress against malaria stalling despite huge investment and implementation of effective interventions across high- and low-burden countries? One explanation is the successful targeting of the ‘low-hanging fruit’: the easiest populations to reach and indoor malaria transmission where there are functioning health systems, while the ‘last mile’ challenges such as difficult-to-reach populations, outdoor-biting vectors and poor quality health services remain largely impervious to standard control strategies [3, 4]. Operational obstacles limit effective delivery of established strategies, including barriers to individual uptake, weak management capacity, as well as insecticide and anti-malarial drug resistance and climate change. Added to the challenges are a limited selection of interventions available for programmes to choose from [1]. While the global malaria community is clear that countries need to shift away from a one-size-fits-all approach, there is a lack of clarity on how programmes can develop tailored, locally appropriate approaches to overcome challenges in malaria control and elimination [5, 6].

How will programmes target residual transmission concentrated among specific groups of individuals who spend time outdoors for work or leisure, or sleep in unprotected structures, as well as individuals who have poor access to health services, because they are migrating, undocumented or are simply living in an area with poor health system effectiveness [7]? How can adequate engagement with affected communities be ensured, to target behaviour change in response to community non-adherence [8]? Here, we propose that district health teams, who are usually responsible for delivery of general and specialist health services, including malaria interventions, can be empowered and trained to become malaria leaders, able to implement solutions to their site-specific malaria transmission patterns and operational challenges. This change would require empowerment and capacity building of the district health teams to make malaria control and elimination decisions in consultation with NMCPs, a radical shift that necessitates deliberate planning and careful assessment. This paper presents a framework for shifting from a one-size-fits-all approach to tailoring and targeting at district level to overcome stalled progress toward malaria control and elimination.

## A framework for district-level leadership and decision making

**Fig. 2:**
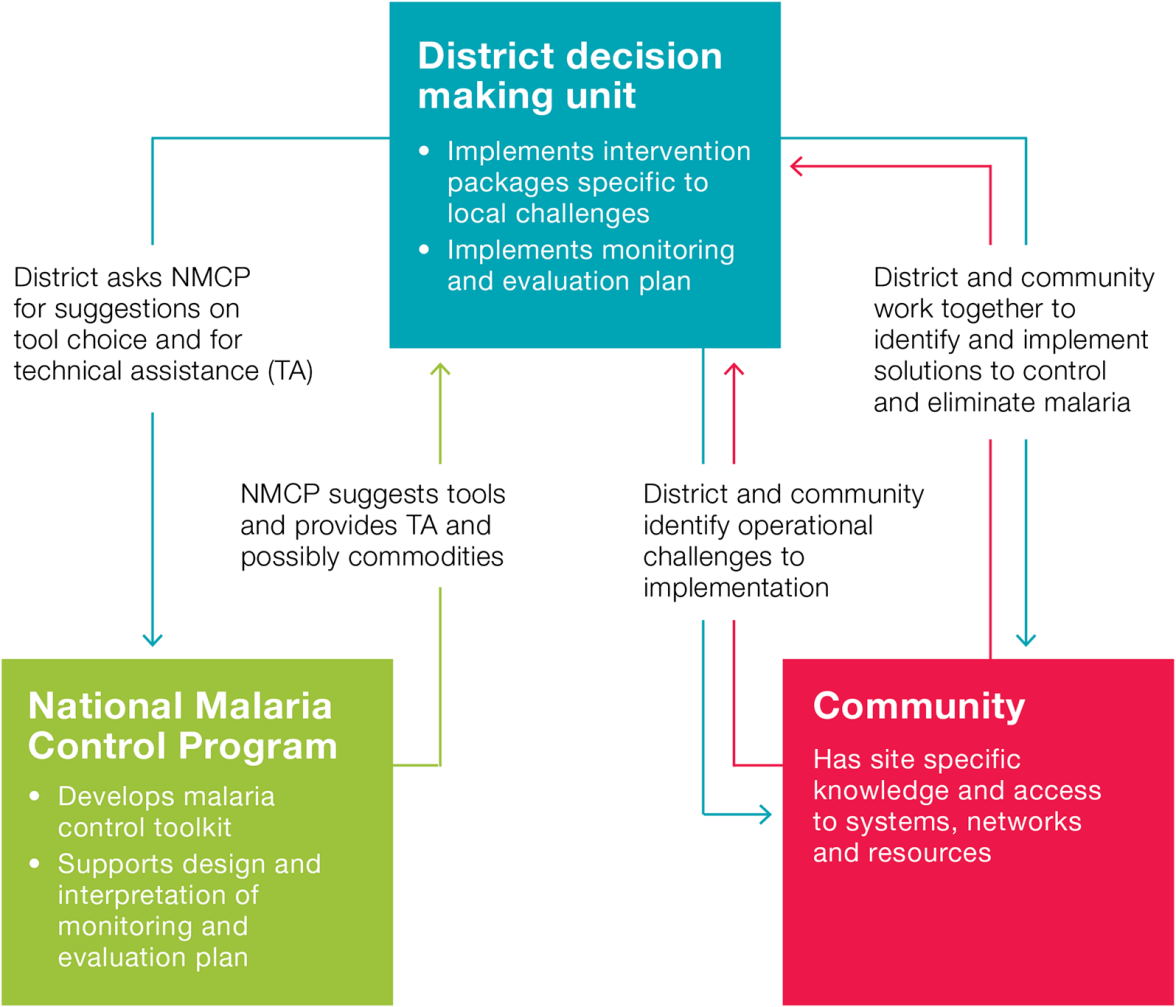
District-level management: a framework of roles and responsibilities for the national malaria control programmes, the district and the community

For the purposes of this paper, national, provincial, and district levels refer to geographical administrative levels 1, 2, and 3 or 4, respectively. A shift from vertical, national-led strategies to district-level decision-making requires changes in the structure, roles and responsibilities of the NMCP, the district and the community. We propose a framework that could enable these changes (Fig. 2), moving away from current practice where NMCPs decide on standard packages of interventions, driven by donor preferences, that are implemented in a ‘top-down’ manner. The proposed role of the NMCP, district and communities in this framework are described below, and elaborated upon in four areas: goal setting and strategy development, monitoring and evaluation, training and mentoring, and financing.

We propose that the NMCP becomes the holder of tools and expertise that can be deployed to solve malaria control problems facing districts and communities. Being in regular contact with districts, the NMCP will provide district health officers with technical guidance, supporting them to establish what is working and what is not, and to develop solutions to new challenges as they arise. The district would be the primary holder of knowledge on malaria transmission patterns, including which communities and groups are at high risk, as well as operational barriers to effective delivery and uptake. Leveraging technical input from NMCP and provinces as described above, districts would work closely with affected communities and other relevant stakeholders to implement a combination of solutions tailored to specific district- or community-level challenges. Local knowledge provided through active community engagement would be key to the success of the proposed framework. Regular, substantive engagement between districts and community leaders would allow for voices across the delivery continuum to be heard and ensure strategies to overcome challenges are built together, motivating end-users to take ownership of malaria control and elimination [8–10].

## Goal setting and strategy development

NMCPs would have periodic meetings with district health officers, perhaps at provincial level, jointly setting district-level elimination goals and determining metrics for inclusion in district health plans. NMCPs would hold districts accountable to these goals and would support districts to decide which intervention packages to implement, comprised of various combinations of tools designed to solve local epidemiologically and socially relevant malaria challenges. As current intervention choices are few in number and do not reflect the breadth of knowledge and newer commodities available to the malaria community, we suggest the current malaria toolkit is expanded to provide information on various commodities and strategies that can be used to address different malaria-related challenges, including plans to ensure community-level buy-in and uptake (see Box 1). Each tool could describe one type of commodity or strategy (examples in Box 1), including information on how to use it and what it is best suited for. New technologies and their combination should be included in the toolkit and evidence for their impact and usefulness gathered through pilot implementation, notwithstanding and in support of evidence from formal trials where available.

Box 1 NMCP toolkit topics The NMCP toolkit must present a variety of options for district health leaders to consider. The list below is not comprehensive, and should be used as an example of the many types of guidance that NMCPs can opt to include.

1. Standard global guidance on widely used strategies:
  Standard and community case management.
  Surveillance (aggregate, case based, human movement, and entomological), data analysis and interpretation.
  LLIN distribution.
  IRS implementation.
  Larval source management.
2. Guidance on more specialized approaches:
  Active surveillance methods.
  Active drug-based strategies (e.g. reactive case detection, focal drug administration, mass drug administration).
  Targeting and reaching specific high-risk populations.
  Partnering with informal private providers where they are the main first point of care.
  Newer commodities (e.g., tafenoquine, new diagnostic tests, spatial repellents, ivermectin).
  New computer-based technologies (e.g. predictive risk maps, mobile phone technology and apps).
3. Guidance on mitigating threats:
  Drug resistance.
  Insecticide resistance.
  Outbreak management.
  Population movement/parasite movement.
4. Process improvement techniques.
  Community engagement models and tools.
  Programme management and quality improvement models.

## Monitoring and evaluation

Once intervention packages for local settings are selected and implemented, the NMCPs and districts would iteratively review whether the packages are working, and identify newly emerging challenges. These reviews would be based on the availability of data and monitoring and evaluation plans developed in collaboration between the NMCP and district.

To facilitate the success of district-level planning and implementation, the NMCP would need to decentralize data management, devolving access to national malaria information systems so that districts have the flexibility and managerial autonomy to make data-driven decisions. This movement is already underway, where Health Management Information System data are becoming increasingly available in formats that are useful for district-level planning. DHIS 2 dashboards and Spatial Decision Support Systems (SDSS) are highly interactive, providing maps, charts, and tables that summarize intervention coverage, malaria case rates, and additional variables of interest depending on what is needed by each cadre in the health system [11–13]. Some SDSSs can predict malaria risk through modelling of weather and other satellite data, helping districts anticipate and prepare for outbreaks and increases in cases [13].

Districts would regularly engage with community representatives and other important local stakeholders, such as military, fishing camps, and traditional healers to identify factors associated with low uptake and/or ineffectiveness of current interventions, and to find feasible solutions together utilizing or adapting existing community structures where possible. Community wisdom would be leveraged to understand why certain interventions may not be working optimally, and community members could co-lead the design and delivery of new operational strategies, unlocking community potential currently under-utilized and under-estimated by the health system. Processes to include communities and stakeholders systematically in public health decision-making are numerous and scaleable [8].

## Training and mentoring

In the shift towards district-led decision-making, the NMCP or relevant training body responsible for capacity improvement would support districts to build the skills needed to identify and prioritize existing and emerging problems, and help districts build capacity to implement the right mix of solutions. The consultations between the NMCP and districts described above form the three critical components of capacity that need to be built. First, to identify drivers of local malaria transmission; second, to develop tailored strategies that target these drivers of transmission including tailored approaches for community engagement; and, third, to measure whether these strategies are working through a simple monitoring and evaluation framework. The NMCP would need to shift these responsibilities to the province or district once requisite skills are developed, and in parallel, provide support for improving district-level leadership, team work and quality management, all of which are critical inputs for health system improvement.

Improving district-level leadership of malaria programmes through training should be a top priority, as the biggest gaps to delivering malaria interventions are operational barriers at the periphery of the health system [1]. In many countries, district health system personnel do not receive management support or training in areas such as health decision-making, strategy design and adaptation, or advocacy to mobilize political support or financial resources. Training programmes that teach, mentor and empower district-level health management teams to effectively gather and optimally use information, motivate their staff and engage local community leaders for their active participation in malaria control and elimination efforts will likely provide tremendous returns on investment for the health system as a whole [14].

## Decentralized financing

The success of this framework would require financial allocations to align with the malaria metrics and targets set at the district level. We suggest that this could best be carried forward using decentralized financing, where the NMCP would provide oversight and procure commodities through national or regional supply chains, and otherwise cede control of budgets to the district. This would enable financial resources to be readily available to districts, such that they can implement interventions more efficiently. Furthermore, districts would have the opportunity to find efficiencies that are invisible to national programmes, for example, integrating supervision and monitoring visits for multiple diseases, incorporating malaria-specific reports into standing meetings, and providing flexible and adaptive funding that could rapidly adjust to changing needs including malaria outbreaks.

Donor financing mechanisms would need to adapt to the new way of implementing malaria control and elimination. In particular, allowing flexible funding to be held at the district or provincial levels to enable a rapid response to changes in the field, such as outbreaks, migration and specific human behaviour of an at-risk group; and, for currently siloed programme activities of vector control, case-management, surveillance, monitoring and evaluation, and social behavioural change communication to mix at the point of intervention delivery.

## Challenges to implementation

Decentralizing decision-making power within malaria programmes to lower-level operational units is a radical proposition in most countries. The change to district-led implementation would require empowering district health management to make decisions and to have control of finances to support their decisions, and ceding some control by the national programme.

Empowering districts to make malaria control and elimination decisions also gives rise to risks. First, weak district leadership may not have the capacity to implement decentralized management and could lead to low-quality malaria control. However, under current structures without efforts to improve district leadership and team function, poorly performing districts exist with little hope of improvement. Second, changes in the health system at the periphery may drive a conflict against central control. Programmes will need to proceed with caution as changes are implemented. Third, giving responsibility to lower levels of the health system may amplify areas where technical capacity is already weak, such as entomology, data analysis and data interpretation. NMCPs and districts will need to ‘ringfence’ resources in anticipation, targeting these identified areas for capacity strengthening, ensuring that training is aimed at lower level health professionals where highly skilled workers are not available.

Not all sub-national units would be capable of moving forward with a decentralized approach. Some well-performing districts and provinces would be able to move towards with this model of working and could act as pilot implementations. Other districts will need substantial improvements in capacity before adoption. Such districts could remain under the guidance of the NMCP until capacity is built within the health system to operate the new model, or development partners could invest in supporting such districts to reach sufficient competence to be able to decentralize decision-making.

## Conclusion

The business-as-usual approach to malaria control and elimination is no longer reaping gains in an environment of ‘flatlined’ funding and more complex, heterogeneous transmission patterns. There is recognition that one-size-fits-all strategies must be abandoned and replaced with demand-driven, problem-based solutions to local operational challenges. Stratification is one step towards this vision, however, stratification will not deal with broader health systems challenges that prevent delivery of chosen anti-malaria strategies, nor will stratification deal with specific community technical challenges, such as varied causes of residual transmission. The district-level approach can support stratification by describing the types of challenges that districts face and their solutions, thus building a more robust strata level toolkit. We suggest that programmes considering shifts towards a district-level approach; use Box 2 as a reference for the steps necessary to change their malaria programme structure, management processes and financing by starting in pilot districts and expanding with experience.

Despite challenges that may result from the programmatic re-orientation we propose, change is needed to overcome stalled progress. Investment in and empowerment of districts will increase their ability to target and deliver quality interventions based on local contextual knowledge. Thailand and The Phillippines demonstrate early successes in implementing district and community-led malaria programmes, a trend we expect to increase in the larger movement towards integrated and decentralized health systems prescribed by the Sustainable Development Goals and Universal Health Coverage (Box 3). This opinion piece raises three areas for further discussion and argument regarding implementation of the district-led approach to malaria control and elimination:

1. How to build sustainable capacity at the district level.
2. What structural changes need to be made at the national level.
3. What changes in donor practices and global guidance need to be made.

If done carefully and deliberately, district decision-making can allow for massive advances in the quality, coverage and efficiency of the health system, particularly at the fringes where high-risk and under-served communities reside, fostering an enabling environment for malaria control and elimination, placing those closer to the front lines in the lead.

Box 2 Steps needed to make the shift to district-level management

1. Provide NMCPs with:
  - Access to and training on a broad malaria control and elimination toolkit.
  - Capacity building in facilitation, listening to the issues at hand, and in problem solving.
  - Broad support from the Ministry of Health to access skills found in other departments such as community engagement.
2. Identify areas suitable for the new approach:
  - Districts with stagnation in progress to malaria goals.
  - Districts where capacity exists or the Ministy of Health is willing to invest to make capacity exist in decision-making and leadership.
  - Flexible finances exist to empower districts.
3. Provide selected districts with:
  - Advocacy to increase local political commitment to the goal.
  - Change/organization management support.
  - Support in data assessment, intervention choices, and monitoring and evaluation.
  - Collaborations between the health system and affected communities to understand malaria transmission and seek feasible solutions to interrupting it.
4. Provide affected communities with a platform to share knowledge on malaria and human behaviour, and seek collaborative solutions to malaria.
5. Hold annual planning meetings, probably at the provincial level, that involve all levels of those involved in the process: the NMCP, the implementation team, community representatives, and other relevant stakeholders.

### Box 3 A shift towards decentralized health systems and successful examples in Thailand and The Philippines

In the past few years, there has been movement toward smaller, sub-national geographical and administrative areas (e.g., provinces, states) applying for malaria-free certification, a process endorsed by WHO but managed independently by each country. Countries with heterogeneous transmission and highly devolved health systems across Asia–Pacific and Latin America have been pursuing progressive sub-national elimination by achieving and certifying malaria elimination province by province. This can be a motivating factor for provinces to support their constituent districts to tackle pockets of ongoing transmission by tailoring interventions to suit the situation of the particular locality. While many countries have assigned sub-national responsibility for implementation of malaria control and elimination programmes, some countries are leading the way by also decentralizing funding and decision-making to the district level.In Thailand, the annual malaria elimination targets of the national strategy are set at the district level. Data from the national malaria information system is used to annually stratify malaria transmission down to the village level, classifying all endemic villages in the country based on the number of active foci. The data are shared with sub-district (*Tambon*) health staff who can appeal to decision-makers to allocate adequate resources from local funding sources to fund the necessary prevention, case management and vector control interventions.

In The Philippines, elected local chief executives (*barangay* captains and mayors) are being engaged to support and lead malaria elimination hand-in-hand with district malaria workers and health officers. Community trust funds have also been established to build a permanent and sustainable community source of funding to supplement the cost of critical malaria control programme activities at the provincial and municipal levels in eliminating provinces [15].

## Data Availability

This paper only contains data and material that is publicly available.

## Abbreviations

ACT: Artemisinin-based combination therapy
IRS: Indoor residual spraying
LLIN: Long-lasting insecticide-treated bed net
NMCP: National Malaria Control Programme
RDT: Malaria rapid diagnostic test
SDSS: Spatial Decision Support System
